# Global inactivation of carboxylesterase 1 (*Ces1/Ces1g*) protects against atherosclerosis in *Ldlr*^−/−^ mice

**DOI:** 10.1038/s41598-017-18232-x

**Published:** 2017-12-19

**Authors:** Jiesi Xu, Yang Xu, Yanyong Xu, Liya Yin, Yanqiao Zhang

**Affiliations:** 1 Department of Integrative Medical Sciences, Northeast Ohio Medical University, Rootstown, OH 44272 USA; 2 State Key Laboratory of Molecular Developmental Biology, Institute of Genetics and Developmental Biology, Chinese Academy of Sciences, Beijing, 100101 China

## Abstract

Atherosclerotic cardiovascular disease is a leading cause of death in the western world. Increased plasma triglyceride and cholesterol levels are major risk factors for this disease. Carboxylesterase 1 (*Ces1/Ces1g*) has been shown to play a role in metabolic control. So far, the role of mouse *Ces1/Ces1g* deficiency in atherosclerosis is not elucidated. We generated *Ces1/Ces1g* ^−/−^ mice. Compared to wild-type mice, *Ces1/Ces1g* ^−/−^ mice had reduced plasma cholesterol levels. We then generated *Ces1g* ^−/−^ *Ldlr* ^−/−^ double knockout (DKO) mice, which were fed a Western diet for 16 weeks. Compared to *Ldlr* ^−/−^ mice, DKO mice displayed decreased plasma cholesterol and TG levels and reduced atherosclerotic lesions. Interestingly, knockdown of hepatic *Ces1/Ces1g* in *Apoe* ^−/−^ mice resulted in hyperlipidemia and exacerbated Western diet-induced atherogenesis. Mechanistically, global inactivation of *Ces1/Ces1g* inhibited intestinal cholesterol and fat absorption and Niemann-Pick C1 like 1 expression, and increased macrophage cholesterol efflux by inducing ATP-binding cassette subfamily A member 1 (ABCA1) and ABCG1. *Ces1/Ces1g* ablation also promoted M2 macrophage polarization and induced hepatic cholesterol 7α-hydroxylase and sterol 12α-hydroxylase expression. In conclusion, global loss of *Ces1/Ces1g* protects against the development of atherosclerosis by inhibiting intestinal cholesterol and triglyceride absorption and promoting macrophage cholesterol efflux.

## Introduction

Cardiovascular diseases (CVDs) are the most prevalent cause of death globally, accounting for about 31% of all global deaths^1,2^. Atherosclerosis is the leading cause of CVDs. Hypercholesterolemia, hypertriglyceridemia and inflammation are common risk factors for atherosclerosis. Liver and intestine play the major role in maintaining plasma lipid and lipoprotein homeostasis by regulating very low-density lipoprotein (VLDL) or chylomicron secretion and lipoprotein uptake, and therefore can regulate the development of atherosclerosis. Macrophages directly participate in the pathogenesis of atherosclerosis by regulating oxidized lipid uptake, cholesterol efflux and inflammation.

Human carboxylesterase 1 (*CES1*) is predominantly expressed in liver and has been shown to have triglyceride (TG) hydrolase activity^3^. Over-expression of human CES1 in macrophages leads to an increase in cholesteryl ester hydrolysis and free cholesterol efflux and attenuation of atherosclerosis in *Ldlr* ^−/−^ mice^4^. In addition, over-expression of human CES1 in the liver enhances reverse cholesterol transport and reduces atherosclerosis in *Ldlr* ^−/−^ mice^5,6^. These data suggest that human CES1 has cholesteryl ester hydrolase (CEH) activity. However, the CEH activity of human CES1 cannot be confirmed by other research groups^7,8^. Mouse *Ces1* gene has multiple isoforms^9^. Mouse *Ces1d* (previously called *Ces3*) and *Ces1g* (previously called *Ces1*) share 78% and 74% amino acid identities with human CES1, respectively. The studies from us and Lehner’s group have previously reported that mouse *Ces1g* has triglyceride hydrolase (TGH) activity^10,11^. Bie *et al*. showed that hepatic deletion of *Ces3/Ces1d* aggravates atherosclerosis in *Ldlr* ^−/−^ mice^12^. In contrast, global loss of *Ces3/Ces1d* is shown to reduce atherosclerosis in *Ldlr* ^−/−^ mice^13^. So far, it is unclear whether loss of mouse *Ces1/Ces1g* has any impact on atherosclerosis.

In this report, we generated global *Ces1/Ces1g* ^−/−^ mice and *Ces1/Ces1g* ^−/−^ *Ldlr* ^−/−^ (DKO) mice. Our data show that loss of *Ces1/Ces1g* in *Ldlr* ^−/−^ mice inhibited the development of atherosclerosis by inhibiting intestinal cholesterol absorption and inducing macrophage cholesterol efflux and bile acid synthesis. Similar to loss of hepatic *Ces3/Ces1d* function, inactivation of hepatic *Ces1/Ces1g* in *Apoe* ^−/−^ mice aggravated the development of atherosclerosis. Our data indicate that *Ces1*/*Ces1g* plays a critical role in the pathogenesis of atherosclerosis.

## Results

### Generation and characterization of *Ces1/Ces1g*^−/−^ mice

**Figure 1:**
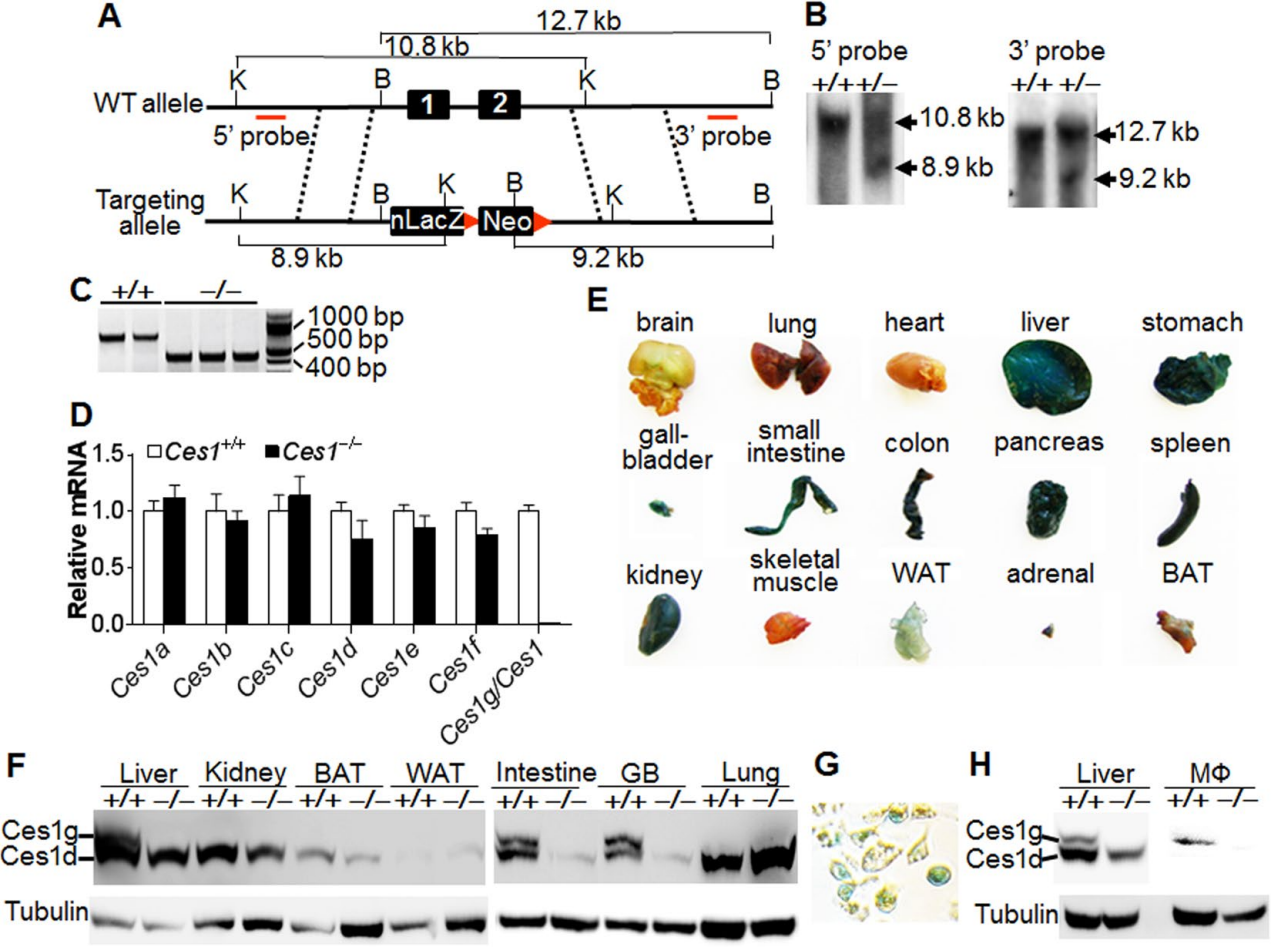
Generation of global *Ces1/Ces1g* ^−/−^ mice and *Ces1/Ces1g* tissue distribution. (**A**) A diagram showing the *Ces1/Ces1g* wild-type (WT) allele and the targeting allele. Exons 1 and 2 are replaced by a nucleus LacZ (nLacZ) cassette and neomycin cassette. K, KpnI. B, BamHI. (**B**) Genomic DNA from WT or heterozygous ES cells was used for Southern blotting using a 5′ probe (left panel) or 3′ probe (right panel). (**C**) Mouse genomic DNA was genotyped by PCR. (**D**) Hepatic mRNA levels of different *Ces1* isoforms were determined (n = 4). (**E**) LacZ staining of various tissues isolated from *Ces1/Ces1g* ^+/−^ mice. (**F**) Hepatic proteins were isolated from 8-weeks-old male control littermates (*Ces1/Ces1g* ^+/+^ mice) or *Ces1/Ces1g* ^−/−^ mice. Protein levels were determined by Western blotting. The top band is Ces1/Ces1g. (**G**) LacZ staining of peritoneal macrophages isolated from *Ces1/Ces1g* ^+/−^ mice. (**H**) Protein levels in the liver (serve as control) or peritoneal macrophages of *Ces1g* ^+/+^ or *Ces1g* ^−/−^ mice were determined by Western blotting. BAT, brown adipose tissue. WAT, white adipose tissue. GB, gallbladder. The Western blots are cropped from larger gels.

We have recently shown that hepatic *Ces1/Ces1g* plays a critical role in maintaining hepatic and plasma lipid homeostasis and is an important TG hydrolase in the liver^11^. To better understand the role of *Ces1/Ces1g* in peripheral tissues, we generated global *Ces1/Ces1g* ^−/−^ mice by replacing *Ces1g* exon 1 and exon 2 with an nLacZ-neomyclin cassette (Fig. 1A). The successful creation of the *Ces1/Ces1g* ^−/−^ mice was confirmed by Southern blotting, PCR genotyping and Western blotting (Fig. 1B–H). Other isoforms of *Ces1* were not affected in the liver of *Ces1/Ces1g* ^−/−^ mice (Fig. 1D; for primer sequences see Supplementary Table 1). LacZ staining of tissues isolated from *Ces1* ^+/−^ mice indicated that *Ces1* was expressed in a number of tissues, including brain, lung, liver, stomach, gallbladder, small intestine, colon, pancreas, spleen, kidney, white adipose tissue (WAT), adrenal and brown adipose tissue (BAT), but not in heart or skeletal muscle (Fig. 1E). Since LacZ staining was not quantitative, we also measured mRNA and protein levels of *Ces1g*. Results from RNA profiling showed that *Ces1/Ces1g* was most abundantly expressed in the liver and to a less extent in other tissues (see Supplementary Fig. 1A). Western blotting results showed that Ces1/Ces1g protein was expressed in the liver, intestine and gallbladder (Fig. 1F), consistent with a previous finding by Quiroga *et al*.^10^. Interestingly, we did not observe Ces1g protein expression in the lung, kidney, WAT or BAT (Fig. 1F), which was likely due to very low expression of Ces1g in these tissues. There was a significant reduction in the expression of Ces1d protein (the faster migrating band, according to Quiroga *et al*.^10^) in the intestine and gallbladder (Fig. 1F). The reason for the reduced Ces1d expression in the intestine or gallbladder is unclear. However, we believe the reduction of Ces1d expression should be secondary to Ces1g ablation as Ces1d expression did not decrease in the liver or lung of *Ces1g* ^−/−^ mice (Fig. 1F). In addition, *Ces1/Ces1g* was also expressed in macrophages, as demonstrated by positive LacZ staining, (Fig. 1G), qRT-PCR (see Supplementary Fig. 1B) and Western blotting assays (Fig. 1H). There was no gross difference in body weight, size or food intake on a chow diet (data not shown).

### *Ces1/Ces1g*^−/−^ mice have reduced plasma cholesterol levels

**Figure 2:**
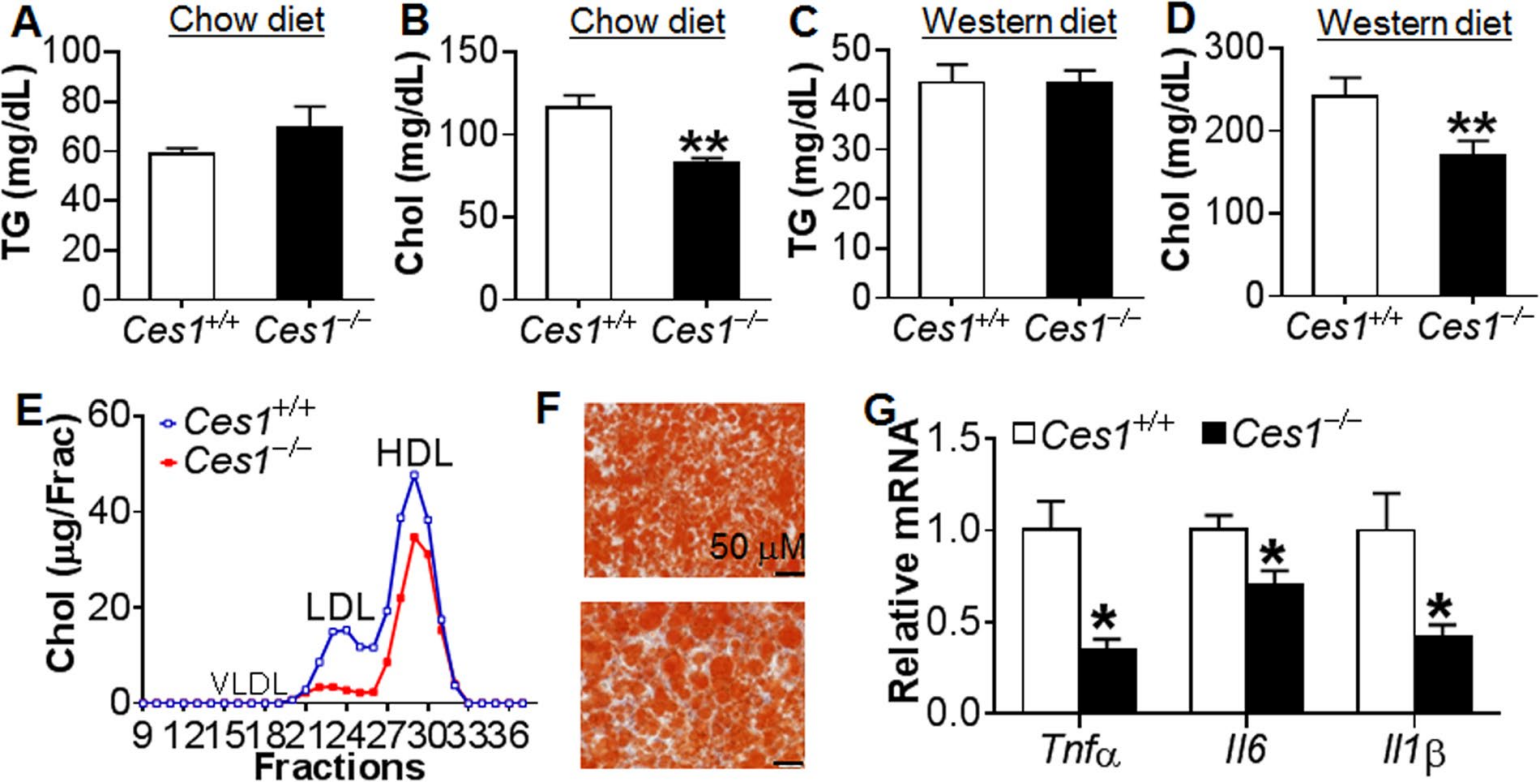
*Ces1/Ces1g* ^−/−^ mice have reduced plasma cholesterol levels. (**A** and **B**) Male *Ces1/Ces1g* ^−/−^ mice and their control littermates (*Ces1/Ces1g* ^+/+^) were fed a chow diet for 6 months (n = 8). Plasma TG (**A**) and cholesterol (**B**) were determined. (C-G) 8-weeks old male *Ces1/Ces1g* ^−/−^ mice and their control littermates (*Ces1/Ces1g* ^+/+^) were fed a Western diet for 16 weeks (n = 8). Plasma TG (**C**) and cholesterol (**D**) levels were determined. FPLC was performed to determine cholesterol distribution in lipoproteins (**E**). Representative liver images of oil red O staining are presented (**F**). Hepatic mRNA levels of inflammatory marker were analyzed (**G**). *P < 0.05, **P < 0.01.

When 8-weeks-old *Ces1/Ces1g* ^−/−^ mice and their control littermates (*Ces1/Ces1g* ^+/+^) were fed a chow diet for 6 months, plasma TG levels were not significantly different between the two genotypes (Fig. 2A), but plasma cholesterol levels were reduced by ~30% in *Ces1/Ces1g* ^−/−^ mice (Fig. 2B). Similar data were obtained when 8-weeks-old *Ces1/Ces1g* ^−/−^ mice and their control littermates were fed a Western diet for 16 weeks (Fig. 2C and D). Analysis of plasma lipoprotein profile by fast protein liquid chromatography (FPLC) indicated that *Ces1/Ces1g* ^−/−^ mice had reduced LDL-C and HDL-C levels (Fig. 2E). In the liver, TG or cholesterol levels were not different between the two genotypes when fed a chow or Western diet (see Supplementary Fig. 2A–D). Oil red O staining showed no difference in neutral lipid accumulation (Fig. 2F). Despite unchanged hepatic lipid levels, *Ces1/Ces1g* ^−/−^ mice had significantly reduced plasma AST and ALT levels (data not shown) and hepatic mRNA levels of inflammatory markers (*Tnfα*, *Il6* and *Il1β*) (Fig. 2G). In addition, Western diet-fed *Ces1/Ces1g* ^−/−^ mice had similar body weight gain and body fat content compared to *Ces1/Ces1g* ^+/+^ mice (see Supplementary Fig. 2E and F).

### Global *Ces1/Ces1g* inactivation reduces atherosclerosis in *Ldlr*^−/−^ mice

**Figure 3:**
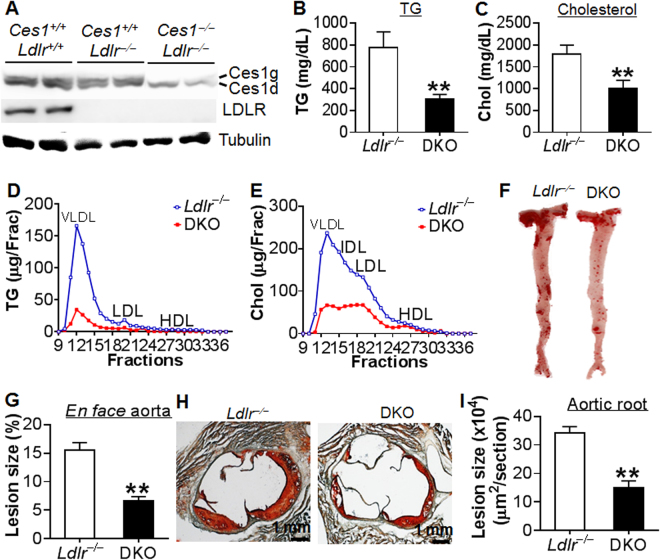
Loss of *Ces1/Ces1g* in *Ldlr* ^−/−^ mice causes hypolipidemia and protects against atherosclerosis. (**A**–**H**) 8-weeks-old male *Ces1* ^−/−^ *Ldlr* ^−/−^ (DKO) mice and control littermates *Ces1* ^+/+^ *Ldlr* ^−/−^ (*Ldlr* ^−/−^) mice were fed a Western diet for 16 weeks (n = 8). Hepatic Ces1/Ces1g and LDLR protein levels were determined (**A**). Plasma TG (B) and cholesterol (**C**) levels were determined. Plasma TG (**D**) and cholesterol (**E**) lipoprotein profiles were analyzed by FPLC. *En face* aortas were stained by oil red O and representative images are shown (**F**). *En face* aorta lesion size was quantified (**G**). Aortic roots were also stained with oil red O and representative images are shown (**H**). Aortic root lesion size was quantified (**I**). **P < 0.01.

The finding that *Ces1/Ces1g* ^−/−^ mice have reduced plasma cholesterol levels led us to investigate whether *Ces1/Ces1g* inactivation affects atherogenesis. We therefore crossed *Ces1/Ces1g* ^−/−^ mice with *Ldlr* ^−/−^ mice to generate *Ces1g* ^−/−^ *Ldlr* ^−/−^ double knockout (*DKO*) mice, which were then fed a Western diet for 16 weeks. The control littermates were *Ces1g* ^+/+^ *Ldlr* ^−/−^ mice. CES1 and LDLR protein levels were shown in Fig. 3A. Compared to *Ces1g* ^+/+^ *Ldlr* ^−/−^ mice, plasma TG and cholesterol levels in DKO mice were lowered by 61% (Fig. 3B) and 44% (Fig. 3C), respectively. In contrast, hepatic TG and cholesterol levels were comparable between control and DKO mice (see Supplementary Fig. 2G and H). FPLC analysis indicated that the reduction in plasma TG and cholesterol levels were attributed to a marked decrease in VLDL-TG (Fig. 3D) and VLDL-C/LDL-C levels (Fig. 3E), respectively. Consistent with markedly improved hyperlipidemia, DKO mice had a ~ 57% reduction in atherosclerotic lesions in both the aortas (Fig. 3F and G) and aortic roots (Fig. 3H and I).

### Liver-specific *Ces1/Ces1g* deficiency exacerbates atherosclerosis in *Apoe*^−/−^ mice

**Figure 4:**
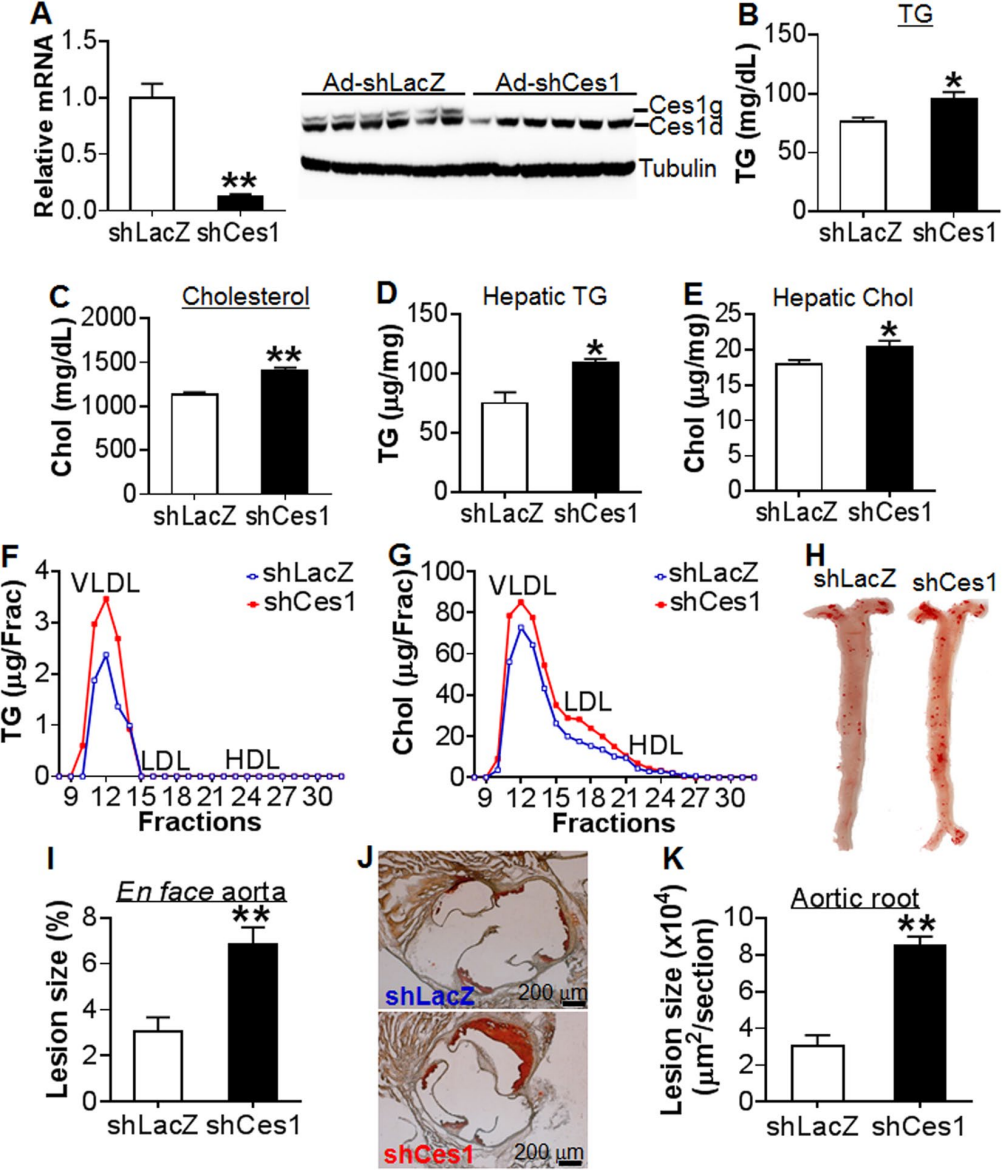
Knockdown of *Ces1/Ces1g* in *Apoe* ^−/−^ mice results in hyperlipidemia and increases atherosclerosis. (**A**–**K**) 12-weeks-old male *Apoe* ^−/−^ mice were fed a Western diet for one week, followed by i.v. injection of Ad-sh*LacZ* and Ad-sh*Ces1* (n = 7). The mice continued to be fed a Western diet for another three weeks. mRNA (left panel) and protein (right panel) levels of *Ces1/Ces1g* were determined three weeks post adenovirus injection (**A**). Plasma levels of TG (**B**) and cholesterol (**C**) as well as hepatic levels of TG (**D**) and cholesterol (**E**) were determined. Plasma TG (**F**) and cholesterol (**G**) lipoprotein profiles were analyzed by FPLC. Representative *en face* aorta images are shown (**H**) and aortic lesion size was quantified (**I**). Representative aortic root images are also shown (**J**) and lesion size was quantified (**K**). *P < 0.05, **P < 0.01.

*Ces1/Ces1g* is abundantly expressed in the liver (Fig. 1). To understand how global inactivation of CES1 ameliorates atherosclerosis, we knocked down hepatic *Ces1/Ces1g* expression in *Apoe* ^−/−^ mice using an adenovirus expressing *Ces1* shRNA (Ad-shCes1). Previously, we showed that Ad-shCes1 can efficiently knock down hepatic *Ces1* mRNA and protein levels by ~95%^11^. The selection of *Apoe* ^−/−^ mice is because these mice can develop spontaneous atherosclerosis even on a regular chow diet. Three weeks after Ad-shCes1 infection, hepatic *Ces1/Ces1g* mRNA and protein levels were reduced by ~90% (Fig. 4A) whereas intestinal CES1g protein levels were unchanged (Supplementary Fig. 3). We did not observe fat malabsorption (data not shown). Similar to what we have reported previously^11^, knock down of hepatic *Ces1/Ces1g* increased plasma TG levels by 21% (Fig. 4B) and cholesterol levels by 20% (Fig. 4C), and also increased hepatic levels of TG (Fig. 4D) and cholesterol (Fig. 4E). FPLC analysis showed that hepatic *Ces1/Ces1g* deficiency increased VLDL-TG (Fig. 4F), VLDL-C and LDL-C (Fig. 4G). Consistent with the latter findings, hepatic *Ces1/Ces1g* deficiency increased atherosclerotic lesions by >50% in both the aortas (Fig. 4H and I) and aortic roots (Fig. 4J and K) of *Apoe* ^−/−^ mice. These data suggest that hepatic *Ces1/Ces1g* is protective against atherosclerosis.

### *Ces1/Ces1g* inactivation reduces intestinal cholesterol and triglyceride absorption

**Figure 5:**
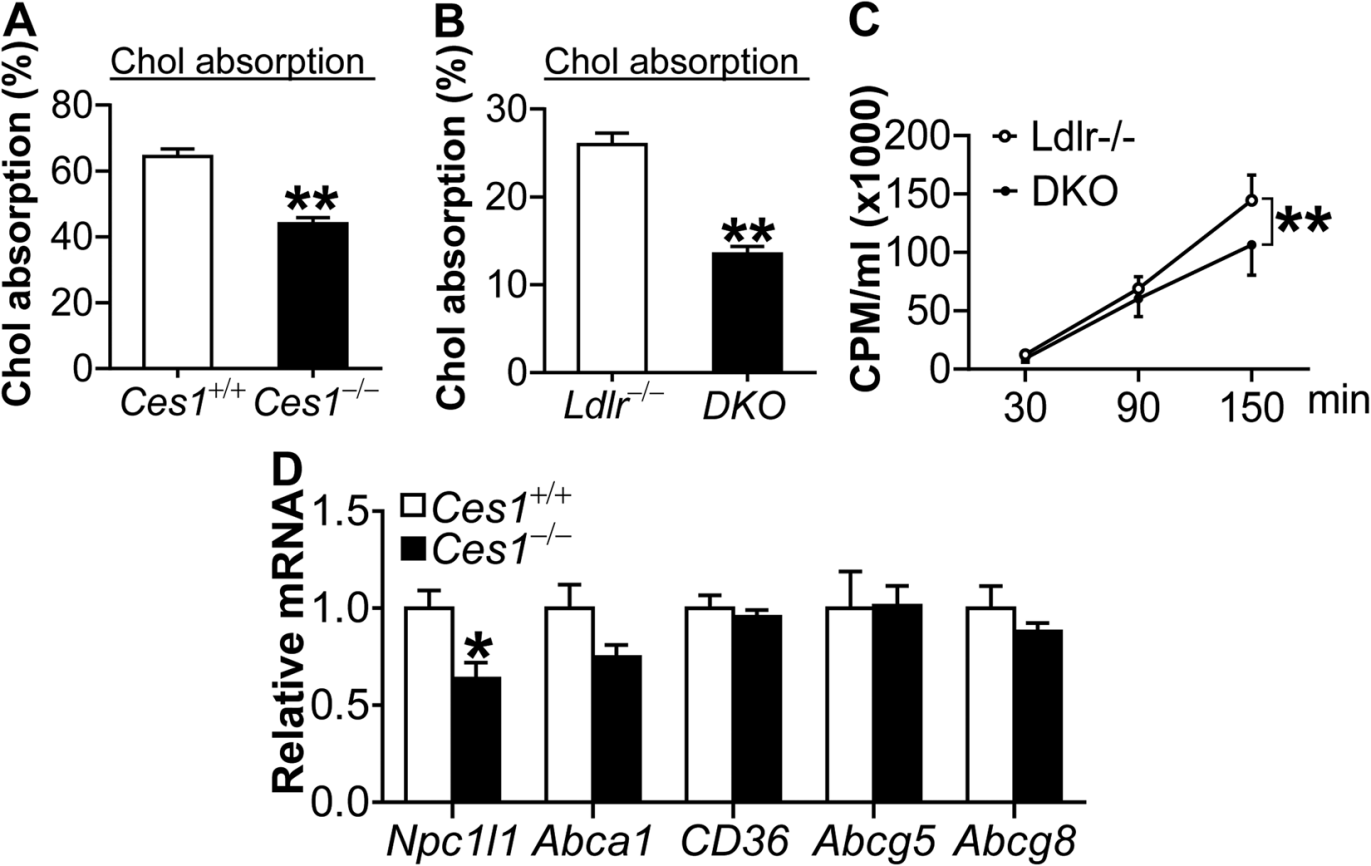
Loss of *Ces1/Ces1g* inhibits intestinal cholesterol absorption. (**A**–**C**) 8-weeks-old male WT and *Ces1/Ces1g* ^−/−^ mice (**A**) or 8-weeks-old male *Ldlr* ^−/−^ and *Ces1* ^−/−^ *Ldlr* ^−/−^ (DKO) mice (**B**) were fed a Western diet for 8 weeks. Cholesterol absorption was then performed (n = 8). Fat absorption was determined in male *Ldlr* ^−/−^ vs DKO mice (n = 8) (**C**). (**D**) mRNA levels in the intestine of 6-months-old male WT mice and *Ces1* ^−/−^ mice were quantified (n = 8). *P < 0.05, **P < 0.01.

The data of Fig. 4 suggest that hepatic *Ces1/Ces1g* is unlikely to contribute to the atheroprotective phenotype in the DKO mice. Since *Ces1/Ces1g* is expressed in intestine (Fig. 1), we investigated whether CES1 inactivation affected cholesterol and/or fat (triglyceride) absorption. The data of Fig. 5A show that *Ces1/Ces1g* ^−/−^ mice had a ~33% reduction in cholesterol absorption. In DKO mice, cholesterol absorption was reduced by 48% (Fig. 5B) and fat (triglyceride) absorption was also significantly reduced (Fig. 5C). Consistent with decreased intestinal cholesterol absorption, the mRNA levels of Nieman-Pick C1-like 1 (*Npc1l1*), a protein that facilities cholesterol transport, were repressed (Fig. 5D). However, other genes involved in cholesterol metabolism (*Abca1*, *Abcg5*, *Abcg8*, *Cd36*) were not significantly changed in *Ces1/Ces1g* ^−/−^ mice vs. control mice (Fig. 5D).

### *Ces1/Ces1g* inactivation induces hepatic CYP7A1 and CYP8B1 expression and bile acid synthesis

**Figure 6:**
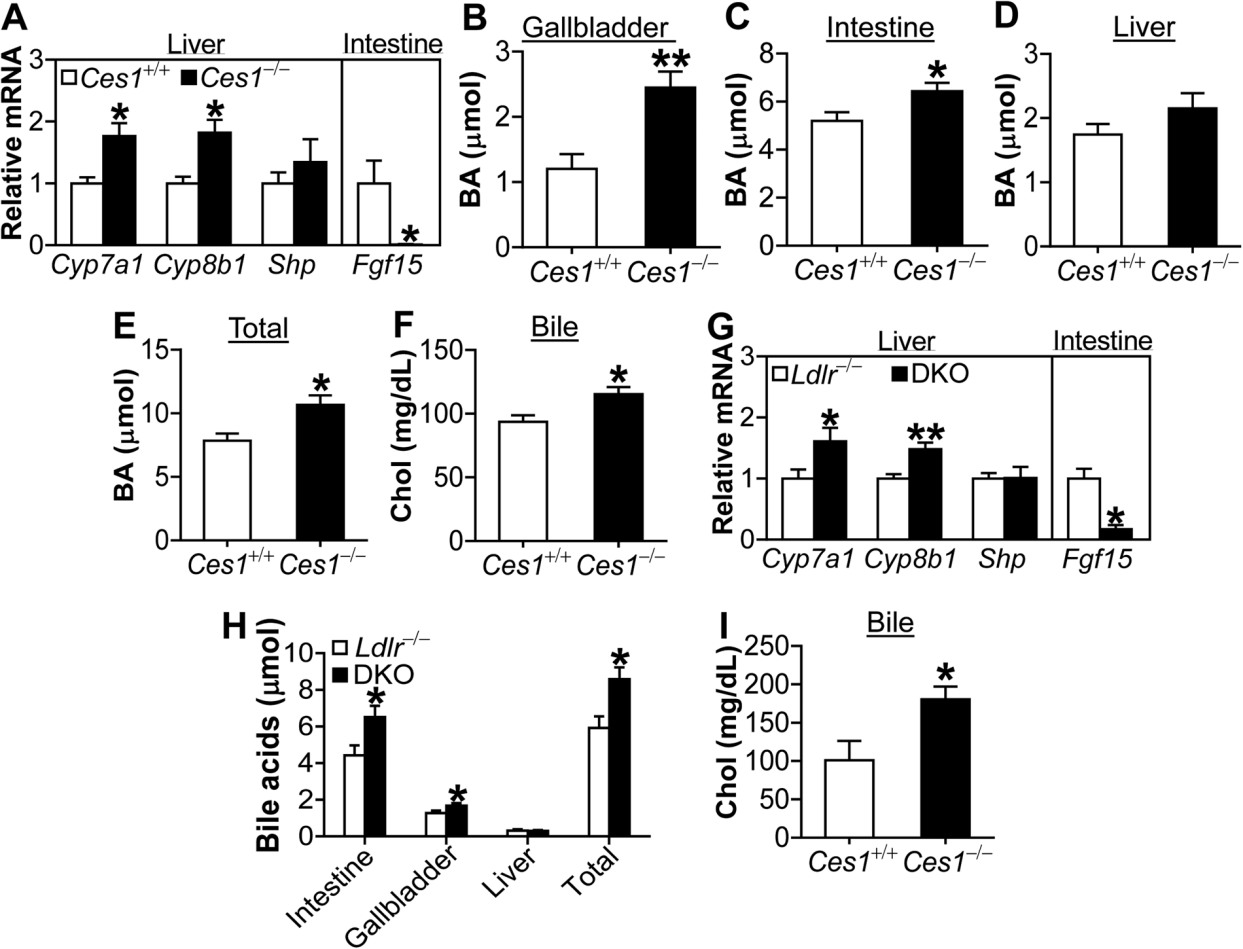
*Ces1/Ces1g* ^−/−^ mice have increased *Cyp7a1* and *Cyp8b1* expression. (**A**–**F**) 6-months-old male chow-fed mice were sacrificed (n = 6). Hepatic and intestinal mRNA levels were quantified by qRT-PCR (**A**). Bile acids (BA) in gallbladder (**B**), intestine (**C**) or liver (**D**) were quantified. Total BA levels were calculated by addition of BA levels in gallbladder, intestine and liver (**E**). Cholesterol concentration in bile was analyzed (**F**). (**G**–**I**) 8-weeks-old male *Ces1* ^−/−^ *Ldlr* ^−/−^ (DKO) mice and *Ldlr* ^−/−^ control littermates were fed a Western diet for 16 weeks (n = 8). Hepatic and intestinal mRNA levels were quantified by qRT-PCR (**G**). Bile acids in liver, gallbladder, intestine as well as total bile acid levels were determined (**H**). Biliary cholesterol levels were also determined (**I**). *P < 0.05, **P < 0.01.

In the liver, cholesterol is converted to bile acids via cholesterol 7α-hydroxylase (CYP7A1) and sterol 12α-hydroxylase (CYP8B1) or secreted directly to the bile. Transgenic expression of CYP7A1 in the liver reduces both HDL-C and non-HDL-C levels^14^ and protects against atherosclerosis^15^. In the liver of *Ces1/Ces1g* ^−/−^ mice, both *Cyp7a1* and *Cyp8b1* mRNA levels were induced by ~1.8 fold (Fig. 6A), which was accompanied by a 99% reduction in fibroblast growth factor 15 (*Fgf15*) in the intestine (Fig. 6A). We did not observe much change in mRNA levels of small heterodimer partner (*Shp*) (Fig. 6A), scavenger receptor class B type 1 (*SR-BI*), ATP-binding cassette subfamily G member 5 (*Abcg5*), *Abcg8*, HMG-CoA reductase (*Hmgcr*), *Apoe* or *Ldlr* (see Supplementary Fig. 4).

Gain- and loss-of-function studies have demonstrated that intestinal FGF15 is an important repressor of hepatic *CYP7A1* and *CYP8B1* expression^16,17^. Consistent with these data, bile acid (BA) levels in gallbladder (Fig. 6B) and intestine (Fig. 6C) were increased whereas BA levels in the liver were unchanged (Fig. 6D). As a result, total BA pool size was increased (Fig. 6E). In addition, biliary cholesterol levels were also increased (Fig. 6F). Similarly, mRNAs levels of hepatic *Cyp7a1* and *Cyp8b1* were elevated and intestinal *Fgf15* level was repressed in DKO mice compared with control littermates (Fig. 6G). In addition, BA levels in gallbladder or intestine were increased in DKO mice, resulting in increased total BA pool size (Fig. 6H). These data suggest that the induction of *Cyp7a1* and *Cyp8b1* may contribute to hypocholesterolemia in *Ces1/Ces1g* ^−/−^ mice.

### Cholesterol efflux is increased in *Ces1/Ces1g* deficient macrophages

**Figure 7:**
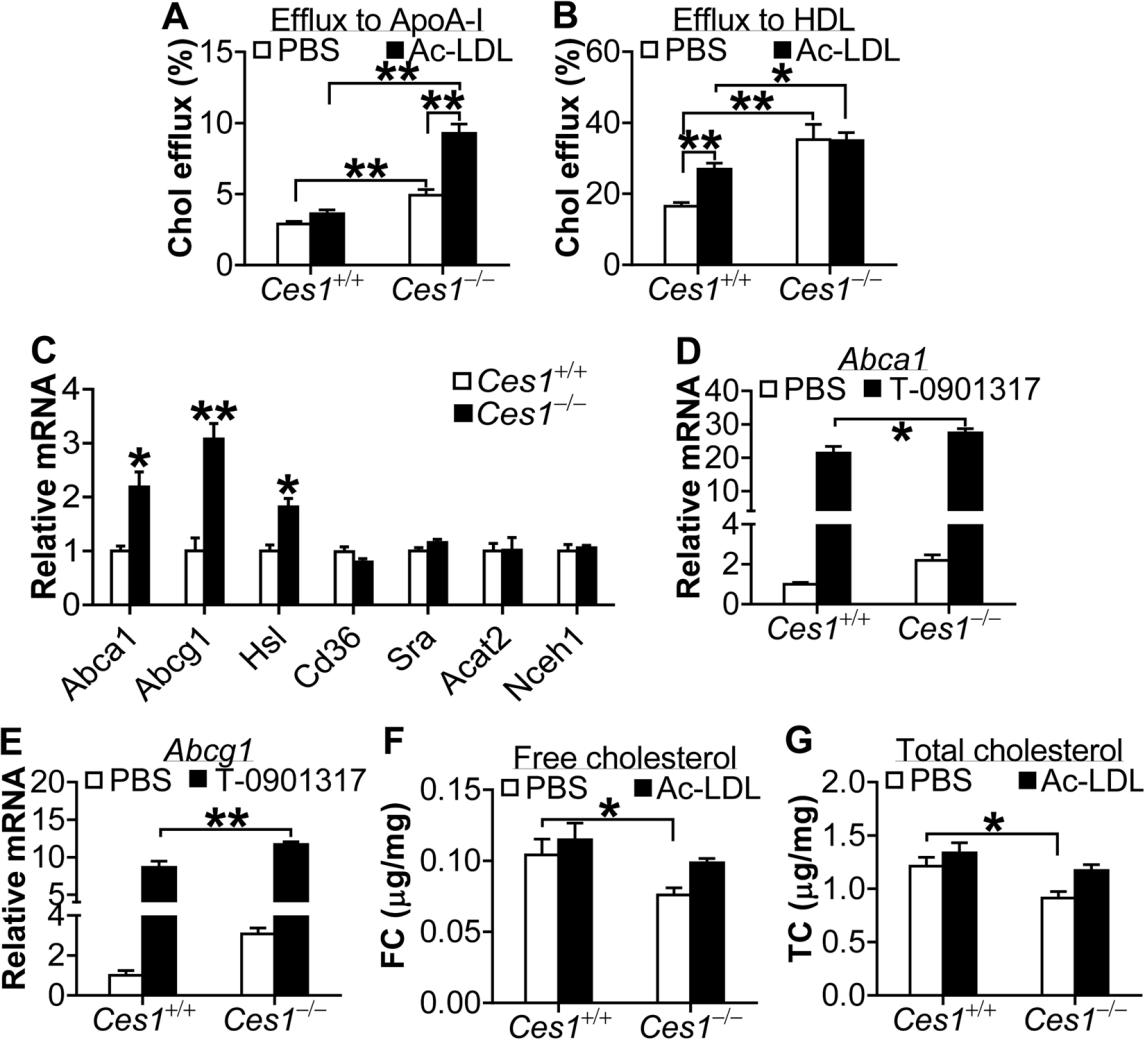
Loss of *Ces1/Ces1g* increases cholesterol efflux from macrophages. (**A** and **B**) Cholesterol efflux to ApoA-I (**A**) or HDL (**B**) was performed in the presence or absence of 25 μg/L Ac-LDL (n = 6). (**C**) mRNA levels were quantified in peritoneal macrophages in the presence of PBS for 24 h (n = 6). (**D** and **E**) Peritoneal macrophages were isolated and treated with 10 μM T-0901317 for 24 h (n = 6). mRNA levels of *Abca1* (**D**) and *Abcg1* (**E**) were quantified. (**F**,**G**) Peritoneal macrophages were treated in the presence or absence of 25 μg/L Ac-LDL for 24 h (n = 5). Intracellular free cholesterol (**F**) and total cholesterol (**G**) were quantified. *P < 0.05, **P < 0.01.

Macrophage cholesterol efflux is the first step in reverse cholesterol transport (RCT). Next, we determined whether loss of *Ces1/Ces1g* in macrophages affected cholesterol efflux. Peritoneal macrophages were isolated from *Ces1/Ces1g* ^+/+^ mice and *Ces1/Ces1g* ^−/−^ mice and then treated with or without acetylated LDL (Ac-LDL). *Ces1/Ces1g* inactivation resulted in a 1.7-fold increase in cholesterol efflux to ApoA-I and this increase was further enhanced in the presence of Ac-LDL (Fig. 7A). In addition, *Ces1* inactivation caused a 2.1-fold increase in cholesterol efflux to HDL (Fig. 7B).

In addition to human CES1, mouse hormone sensitive lipase (Hsl)^7,18^ and neutral cholesterol ester hydrolase 1 (NCEH1)^8^ are reported to promote hydrolysis of cholesterol esters in macrophages. ATP-binding cassette subfamily A member 1 (ABCA1) and ABCG1 are the major transporters that efflux free cholesterol to ApoA-I or HDL, respectively^19^. *Ces1/Ces1g* inactivation significantly increased *Hsl* but not *Nceh1* mRNA levels (Fig. 7C). *Ces1/Ces1g* inactivation in macrophages also caused 2- and 3-fold increase in *Abca1* and *Abcg1* mRNA levels, respectively (Fig. 7C). In contrast, *Ces1/Ces1g* inactivation had no much effect on scavenger receptor class A (*Sra*), fatty acid translocase (*Cd36*) or acetyl-Coenzyme A acetyltransferase 2 (*Acat2*) (Fig. 7C and see Supplementary Fig. 5A–C). T-0901317, a liver X receptor agonist, drastically induced *Abca1* (Fig. 7D) and *Abcg1* (Fig. 7E) mRNA levels in wild-type macrophages, and this induction was further increased in *Ces1/Ces1g* ^−/−^ macrophages (Fig. 7D and E), suggesting that *Ces1/Ces1g* inactivation may lead to increased production of endogenous LXR agonists (oxysterols). In the absence of Ac-LDL treatment, the intracellular free cholesterol and total cholesterol contents were decreased in *Ces1/Ces1g* ^−/−^ macrophages compared with wild-type macrophages, but this decrease became less pronounced when macrophages were treated with Ac-LDL (Fig. 7F and G). Taken together, these data suggest that *Ces1/Ces1g* inactivation increases macrophage cholesterol efflux by inducing both ABCA1 and ABCG1.

### *Ces1/Ces1g* inactivation drives M2 macrophage polarization

**Figure 8:**
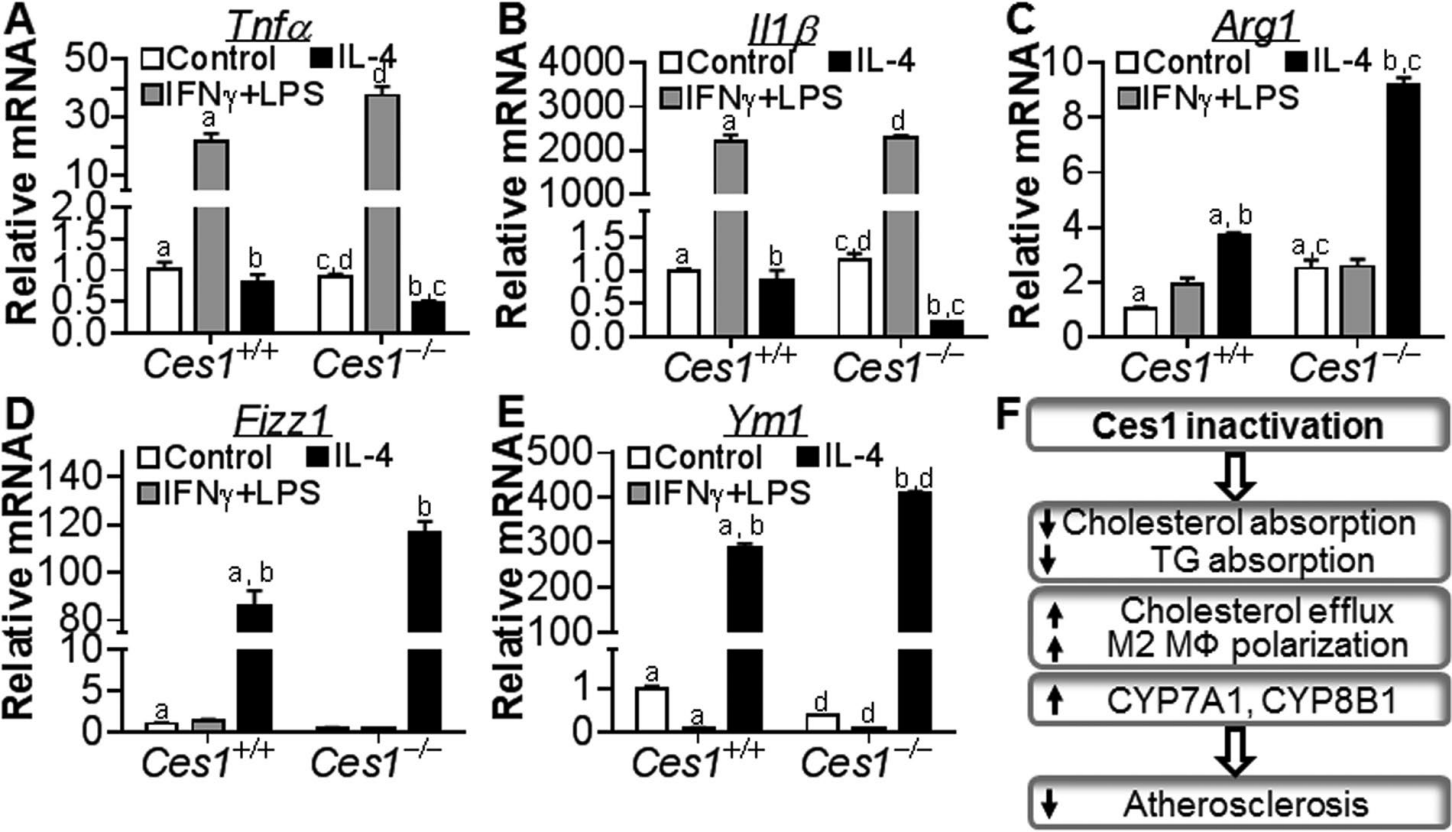
Loss of *Ces1/Ces1g* promotes M2 macrophage polarization. (**A**–**E**) Peritoneal macrophages were isolated from wild-type or *Ces1/Ces1g* ^−/−^ mice and subjected to induction to M1 or M2 macrophages (n = 4). mRNA levels were determined by qRT-PCR. (**F**) Mechanism for Ces1/Ces1g inactivation to repress atherosclerosis. ^a,b,c, or d^ *P* < 0.05.

Recent evidence links inflammatory M1 macrophages to atherosclerotic progression and anti-inflammatory M2 macrophages to atherosclerotic regression^20^. M0 macrophages were treated with interferon γ (IFNγ) plus lipopolysaccharide (LPS) or interleukin 4 (IL-4) to induce M1 or M2 macrophages, respectively. Both tumor necrosis factor α (*Tnfα*) (Fig. 8A) and *Il1β* (Fig. 8B) were markedly induced in both *Ces1/Ces1g* ^+/+^ and *Ces1/Ces1g* ^−/−^ M1 macrophages (grey bars). In contrast, both *Tnf*α and *Il1β* were significantly reduced in *Ces1/Ces1g* ^−/−^ M2 macrophages (black bars) (Fig. 8A and B).

As expected, *Ces1/Ces1g* ^+/+^ M2 macrophages had increased levels of arginase 1 (*Arg1*) (Fig. 8C), resistin like alpha (Retnla/Fizz1) (Fig. 8D) and chitinase-like 3 (Chil3/Ym1) (Fig. 8E), all of which are markers for M2 macrophages. *Ces1/Ces1g* inactivation further increased *Arg1*, *Fizz1* and *Ym1* expression (Fig. 8C–E). In addition, Inactivation of *Ces1/Ces1g* increased the basal levels of *Arg1* (Fig. 8C). Consistent with the *ex vivo* data, in the lesions of *Ces1g* ^−/−^ *Ldlr* ^−/−^ mice vs. *Ces1g* ^+/+^ *Ldlr* ^−/−^ mice, *Abca1*, *Arg1* and *Mrc1* mRNA levels were significantly increased (see Supplementary Fig. 6). Together, these data suggest that *Ces1/Ces1g* inactivation promotes M2 macrophage polarization.

## Discussion

The studies on the role of the carboxylesterase family in regulating lipid metabolism are challenged by the structural and functional similarities shared by their family members^9^. Mouse *Ces3/Ces1d* and *Ces1/Ces1g* share high amino acid identities with human CES1, and both *Ces1d* and *Ces1g* have been reported to play roles in lipid metabolism^11,21^. *Ces3/Ces1d* and *Ces1/Ces1g* share 76% amino acid identity and have the similar protein size, but display different tissue expression profiles. Global or liver-specific loss of *Ces3/Ces1d/TGH* is shown to improve blood lipids by attenuating VLDL secretion^22,23^. *Ces3/Ces1d/TGH* knockout mice are reported to have increased energy metabolism and glucose tolerance^23^. In contrast, loss of *Ces1/Ces1g/Es-x* is reported to induce obesity and hyperlipidemia^10^. In addition, loss of *Ces3/Ces1d/TGH* attenuates atherosclerosis in *Ldlr* ^−/−^ mice, but liver specific loss of *Ces3/Ces1d/TGH* promotes atherosclerosis in *Ldlr* ^−/−^ mice^12,13^. Similar results are found in this study when we inactive *Ces1/Ces1g* in *Ldlr* ^−/−^ mice or *Apoe* ^−/−^ mice. In this report, we provide the first evidence demonstrating that global *Ces1/Ces1g* inactivation markedly attenuates the development of atherosclerosis by reducing intestinal cholesterol absorption, promoting macrophage cholesterol efflux and M2 macrophage polarization, and inducing bile acid synthesis (Fig. 8F) whereas loss of hepatic *Ces1/Ces1g* aggravates the development of atherosclerosis.

Previously, Lehner group show that female, 5–6 months old *Ces1/Ces1g/Es-x* ^−/−^ mice are obese^10^. We do not see any difference in body weight or adiposity between male *Ces1* ^+/+^ mice and male *Ces1g* ^−/−^ mice that are fed either a chow diet or high fat diet. We realize that we may have used mice with different sex (male vs female) and background. Lehner group also reports that *Ces1/Ces1g/Es-x* ^−/−^ mice have increased secretion of chylomicron and decreased chylomicron clearance^24^. We find that loss of *Ces1/Ces1g* ^−/−^ leads to decreased TG and cholesterol absorption in the intestine. Interestingly, in our *Ces1g* ^−/−^ mice intestinal Ces1d expression is also reduced, which may contribute to the reduced fat absorption and overall lipid homeostasis.

In our global *Ces1/Ces1g* ^−/−^ mice, we do not see any change in the levels of hepatic neutral lipids. Previously, we show that liver-specific knockdown of *Ces1/Ces1g* causes hepatic triglyceride accumulation and increased VLDL secretion and plasma lipids^11^. In global *Ces1/Ces1g* ^−/−^ mice, the reduction in intestinal fat and cholesterol absorption may be sufficient to counteract the increased lipogenic activity in the liver, thus resulting in reduced plasma cholesterol levels and unchanged hepatic lipids.

Reverse cholesterol transport begins with macrophage cholesterol efflux to the acceptors ApoA-I or HDL by ABCA1 or ABCG1. Prior to cholesterol efflux, cholesterol esters are hydrolyzed by cholesterol ester hydrolase. Loss-of-function studies have established both HSL and NCEH1 as important cholesterol ester hydrolases in macrophages^7,8^. Our data show that *Ces1/Ces1g* is present in peritoneal macrophages. So far, it is unclear whether mouse *Ces1/Ces1g* has cholesterol ester hydrolase activity. Interestingly, loss of *Ces1/Ces1g* in macrophages induces *Hsl*, *Abca1 and Abcg1* expression. HSL over-expression in macrophages is reported to increase cholesterol ester hydrolysis^18^. The induction of *Abca1* and *Abcg1* suggests that intracellular oxysterols may be increased in *Ces1/Ces1g* ^−/−^ macrophages. Indeed, LXR activation is further enhanced in *Ces1/Ces1g* ^−/−^ macrophages. In the future, we will determine whether oxysterols and other lipid metabolites are changed in *Ces1/Ces1g* ^−/−^ macrophages.

In addition to regulating cholesterol efflux, our data also show that *Ces1/Ces1g* inactivation promotes M2 macrophage polarization. M2 macrophages are anti-inflammatory and play an important role in regression of atherosclerosis^20^. Although the mechanism underlying the regulation of macrophage polarization remains to be determined, our data indicate that loss of *Ces1/Ces1g* in macrophages has two beneficial effects − promoting cholesterol efflux and inhibiting inflammation.

Liver is one of the most important organs regulating the pathogenesis of atherosclerosis. Our data show that *Ces1/Ces1g* knockdown in the liver promotes the development of atherosclerosis in *Apoe* ^−/−^ mice whereas global loss of *Ces1/Ces1g* protects against atherosclerosis in *Ldlr* ^−/−^ mice. Over-expression of hepatic *Ces1g* produces less atherogentic lipoproteins^11,25^, which may account for the atheroprotective effect of hepatic *Ces1g*. Since we used mice with different genetic background (*Apoe* ^−/−^ mice versus *Ldlr* ^−/−^ mice), we are not able to reach a firm conclusion at this point that hepatic *Ces1/Ces1g* deficiency is not part of the mechanism underlying the atheroprotective effect found in DKO mice. However, *Apoe* ^−/−^ mice and *Ldlr* ^−/−^ mice are both widely used for atherosclerosis study and the results achieved using these mouse lines are often very similar. The reduced intestinal TG and cholesterol absorption plays an important role in atheroprotection in DKO mice. In addition, hepatic *Cyp7a1* and *Cyp8b1* are induced in *Ces1/Ces1g* ^−/−^ mice likely as a result of inhibition of intestinal *Fgf15*. Since *Cyp7a1* and *Cyp8b1* are key enzymes in cholesterol catabolism in the liver, the induction of *Cyp7a1* and *Cyp8b1* may also contribute to the atheroprotective effect of *Ces1/Ces1g* inactivation.

In summary, we demonstrate that global inactivation of *Ces1/Ces1g* ameliorates the development of atherosclerosis by inhibiting intestinal cholesterol absorption, promoting macrophage cholesterol efflux and M2 macrophage polarization, and inducing bile acid production. In contrast, inactivation of hepatic *Ces1/Ces1g* aggravates the development of atherosclerosis likely by producing atherogenic lipoproteins^11^.

## Methods

### Mice and diet

To generate *Ces1/Ces1g* ^−/−^ mice, the *Ces1* (*Ces1g*) targeting vector (see Fig. 1) was electroporated into G4 ES cells (129/B6 background; Samuel Lunenfeld Research Institute at Mount Sinai Hospital, Toronto, Canada). After screening by PCR and Southern blotting, positive ES clones were injected into blastocysts from C57BL/6 mice, which were then implanted into pseudopregnant foster mothers in the Transgenic Mouse Facility in University of California, Irvine. Genotyping was performed using primers 5′-CTCAGAGGTTAGCCAGCTGGGAGGA-3′ (forward), 5′-GGCCTTCAGACAGGGAAAAGCTTTG-3′ (reverse, WT, 720 bp) and 5′-TAGCAGCCAGTGGGGTTCTCAGTG-3′ (reverse, mutant, 450 bp). *Ces1/Ces1g* ^+/−^ mice were back-crossed with C57BL/6 J mice for 6 generations to get 99.3% C57BL/6 background before self-crossing to generate *Ces1/Ces1g* ^−/−^ mice and *Ces1/Ces1g* ^+/+^ mice (control), or crossing with *Ldlr* ^−/−^ mice (Jackson Laboratory, Maine, USA) to generate *Ces1/Ces1g* ^−/−^ *Ldlr* ^−/−^ mice and *Ces1/Ces1g* ^+/+^ *Ldlr* ^−/−^ mice (control). *Apoe* ^−/−^ mice were purchased from the Jackson Laboratory (Maine, USA). All the mice were male and were fed a chow diet or a Western diet containing 42% kcal fat and 0.2% cholesterol (TD.88139, Envigo, Wisconsin, USA) for up to 16 weeks, starting from 8 weeks old. All the mice were fasted for 5–6 hours prior to euthanization for samples (tissue, blood, etc) collection. All the animal studies have been approved by the Institutional Animal Care and Use Committee at Northeast Ohio Medical University. All the experiments were performed in accordance with the relevant guidelines and regulations.

### RNA isolation and quantitative real-time PCR

Total RNA was isolated using TRIzol Reagent (Life Technologies, NY). mRNA levels were determined by quantitative reverse-transcription polymerase chain reaction (qRT-PCR) on a 7500 real-time PCR machine from Applied Biosystems (Foster City, CA). Relative mRNA levels were calculated using the comparative cycle threshold (*Ct*) method and were normalized to the values of 36B4 mRNA levels.

### Southern and Western blotting

Southern blotting was performed as described^26^. Western blotting was performed using a CES1 antibody (Cat # ab45957) or Tubulin antibody (Cat # ab4074) from Abcam (Cambridage, MA) or LDLR antibody from Novus Biologicals (Cat # NBP1-06709, Littleton, CO).

### Lipid analysis

Approximately 100 mg liver was homogenized in methanol and lipids were extracted in chloroform/methanol (2:1 v/v). Hepatic and plasma triglyceride and cholesterol levels were quantified using Infinity reagents from Thermo Scientific (Waltham, MA). For analyzing plasma lipoprotein levels, FPLC was performed as described^11^. In brief, 100 μL plasma was injected into the BioLogic DuoFlow QuadTec 10 system (Bio-Rad, Hercules, CA). Lipoproteins were run at 0.5 mL/min in a buffer containing 0.15 M NaCl, 0.01 M Na_2_HPO4, 0.1 mM EDTA, pH7.5, and separated on a Superose 6 10/300 GL column (GE Healthcare). A 500 μL sample per fraction was collected for analyzing lipid levels using Infinity reagents.

### β-galactosidase (LacZ) staining

Peritoneal macrophages or tissues were washed with PBS, fixed with a fixative solution containing 0.5% glutaraldehyde, washed with PBS and stained with a solution containing 1 mg/ml X-gal at 37 °C.

### Peritoneal macrophage isolation

Wild-type and *Ces1/Ces1g* ^−/−^ mice were injected with 1 mL of 3% (w/v) brewer thioglycollate medium into their peritoneal cavity. Four days later, mice were euthanized. The outer skin of the peritoneum was cut with gentle and pulled back to expose the inner skin lining the peritoneal cavity. Dulbecco’s Modified Eagle Medium (DMEM) medium with 10% fetal bovine serum (FBS) was injected into the peritoneal cavity using a 21.5 G needle, followed by gently massage of the peritoneum to dislodge any attached cells into the medium. The cells were collected into a 5-ml syringe. DMEM medium was injected 1–2 more time to retrieve more cells. Cell suspension was spun at 1500 rpm, resuspended and cultured in 6-well plates.

### Intestinal fat absorption

After overnight fasting, mice were injected intravenously with 500 mg/kg of Triton WR1339. Immediately after the Triton injection, mice were given an intragastric 200 μL olive oil containing 7 μCi of [^3^H]triolein (Perkin Elmer). Blood samples were drawn via retro-orbital bleeding at indicated time points after administration of [^3^H]triolein. The amount of ^3^H in plasma was determined using a liquid scintillation counter.

### Intestinal cholesterol absorption

Intestinal cholesterol absorption was determined by a dual-isotope plasma ratio method^27^. Briefly, mice were intravenously injected with 2.5 μCi ^3^H-cholesterol in Intralipid (Sigma, St Louis, MO), immediately followed by oral gavage of 1 μCi ^14^C-cholesterol in median-chain triglycerides (MCT oil, Mead Johnson, Evansville, IN). Mice were returned to cage with free access to food and water. After 72 h, blood samples were collected and the radioactivity of ^14^C and ^3^H were determined by scintillation counting. Intestinal cholesterol absorption was determined as the ratio of ^14^C/^3^H in 1 ml of plasma.

### Cholesterol efflux

Cholesterol efflux was performed as described^28,29^. Peritoneal macrophages from wild-type and *Ces1* ^−/−^ mice were isolated and incubated in DMEM containing 0.2% bovine serum albumin (BSA). Cells were then incubated with acetylated LDL (50 μg/mL) and [^3^H] cholesterol (1 μCi/mL) for 24 hours. After 24 h, cells were washed twice with PBS, and further incubated in DMEM containing 0.2% BSA for 2–4 hours. Cholesterol efflux was performed in the presence of fresh DMEM containing 0.2% BSA, 50 μg/mL of HDL, ApoA-I or Ac-LDL. After 4-h incubation, the radioactivity in the medium and the cell lysate was measured. The percent efflux was calculated as (medium dpm)/(cell dpm + medium dpm) × 100%.

### Bile acid analysis

Bile acids in gallbladder, liver and intestine were extracted with 95% ethanol overnight, followed by 80% ethanol for 2 hours and methanol/chloroform (2:1) for 2 hours at 50 °C. Total bile acids were determined using a bile acid assay kit (Diazyme, San Diego, CA).

### Body composition analysis

Body fat mass and lean mass were measured by Echo-MRI (EchoMRI, LLC, Houston, TX).

### Atherosclerosis study

The aorta, including the ascending arch, thoracic and abdominal segments were dissected, gently cleaned of the adventitia and stained with Oil Red O as described previously^30,31^. In addition, the aortic roots were collected from the base of the heart including the atria and embedded in optimal cutting temperature (OCT) compound. Sections (5 μm) were obtained every 50 μm from the base of the aortic leaflets to 400 μm above. After staining with Oil Red O, images were captured with a microscope, and the lesion area for each aortic ring was analyzed using Image Pro (Media Cybernetics).

### Statistical Analysis

The data were analyzed using unpaired Student *t* test and ANOVA (GraphPad Prisim, CA). All values were expressed as mean ± SEM. Differences were considered statistically significant at *P* < 0.05.

### Data availability

All the data are available upon request.

## Electronic supplementary material

Supplementary Information
