# Supplementary material for: Global inactivation of carboxylesterase 1 (Ces1/Ces1g) protects against atherosclerosis in Ldlr−/− mice
Source: Sci Rep. 2017 Dec 19;7:17845. doi: 10.1038/s41598-017-18232-x (PMC5736751; doi:10.1038/s41598-017-18232-x)

**Supplementary Information**

# Global inactivation of carboxylesterase 1(*Ces1/Ces1g*) protects against atherosclerosis in *Ldlr*$^{-/-}$ mice

Jiesi Xu [1,2], Yang Xu [1], Yanyong Xu [1], Liya Yin [1] and Yanqiao Zhang [1*]

[1] Department of Integrative Medical Sciences, Northeast Ohio Medical University, Rootstown, OH 44272, USA

[2] State Key Laboratory of Molecular Developmental Biology, Institute of Genetics and Developmental Biology, Chinese Academy of Sciences, Beijing 100101, China

Corresponding author:

Yanqiao Zhang, MD

Department of Integrative Medical Sciences

Northeast Ohio Medical University

Phone: (330) 325-6693

Fax: (330) 325-5978

Email: yzhang@neomed.edu

**Supplementary Table1. qPCR primers for mouse *Ces1a-1g*.**

**Supplementary Figure 1. *Ces1/1g* tissue distribution and relative mRNA levels of *Ces1a-1g* genes in macrophages of wild-type or *Ces1/Ces1g*$^{-/-}$ mice**. (A) Hepatic *Ces1/Ces1g* mRNA levels in different tissues of male, 12-weeks-old wild-type mice were determined (n=4). (B) Peritoneal macrophages were isolated from 12-weeks-old male wild-type or *Ces1/Ces1g*$^{-/-}$ mice (n=4). mRNA levels of *Ces1a-1g* were determined. ** *P*<0.01

**Supplementary Figure 2. Global loss of *Ces1/Ces1g* does not affect hepatic TG or cholesterol levels or body fat content.** (A and B) 6-months-old wild-type and *Ces1/Ces1g*$^{-/-}$ male mice were fed a chow diet (n=6). Hepatic TG (A) and cholesterol (B) levels were determined. (C-F) 8-weeks-old male wild-type and *Ces1/Ces1g*$^{-/-}$ mice were fed a Western diet for 16 weeks (n=8). Hepatic TG (C) and cholesterol (D) levels were measured. Body weight was monitored (E) and fat composition was determined by the end of the study (F). (G and H) Male *Ldlr*$^{-/-}$ mice and DKO were fed a Western diet for 16 weeks (n=8). Hepatic TG (G) and cholesterol (H) levels were determined.

**Supplementary Figure 3. Intestinal mRNA levels in *Apoe*$^{-/-}$ mice infected with Ad-shLacZ or Ad-shCes1.** 12-weeks-old male *Apoe*$^{-/-}$ mice were fed a Western diet for one week, followed by i.v. injection of Ad-sh*LacZ* and Ad-sh*Ces1* (n=7). The mice continued to be fed a Western diet for another three weeks. Intestinal protein levels were quantified by Western blotting. * *P*<0.05

**Supplementary Figure 4. Effect of *Ces1/Ces1g* inactivation on gene expression in chow-fed mice.** mRNA levels were quantified in 6-months-old chow-fed mice (n=6). * $P<0.05$

**Supplementary Figure 5. Effect of *Ces1/Ces1g* inactivation on gene expression in macrophages.** (A-C) mRNA levels were quantified in peritoneal macrophages in the presence or absence of 25 μg/ml Ac-LDL for 24 h (n=6). * $P<0.05$, ** $P<0.01$

**Supplementary Figure 6. mRNA levels in the lesions of *Ldlr*$^{-/-}$ mice vs. DKO mice.** 8-weeks-old *Ldlr*$^{-/-}$ mice and *Ces1/Ces1g*$^{-/-}$*Ldlr*$^{-/-}$ (DKO) mice were fed a Western diet for 16 weeks (n=8). mRNA levels in the atherosclerotic lesions were determined. * $P<0.05$

**Supplementary Table 1. qRT-PCR primers for *Ces1a-1g***

| | |
|---|---|
| Ces1a_Forward | TGGCTTCCCTCAACACTTG |
| Ces1a_Reverse | GCGGATCCTTCTAAGCTGATG |
| Ces1b_Forward | TCCTAGAGTTCCTGTCCTTCC |
| Ces1b_Reverse | CTTTCCCAGGATTTTGCCTTG |
| Ces1c_Forward | ATCCTGCCTCTCAAGATTTCG |
| Ces1c_Reverse | GCTAGTCCATTATAGGGTGATCG |
| Ces1d_Forward | AGATAGCTCAGAGACCCACAG |
| Ces1d_Reverse | ATACTTCCCCAGGACTTTGC |
| Ces1e_Forward | TCTTGGATCTCTGAGGTTTGC |
| Ces1e_Reverse | ACTATTTGCCCTGTCACTGG |
| Ces1f_Forward | TTGGATCCCTGAGGTTTGC |
| Ces1f_Reverse | CTGTCCTCTAGCTGCATCTTG |
| Ces1g_Forward | ACACCTCACTAGACCCCAG |
| Ces1g_Reverse | ATACTTCCCCAGGACTTTGC |

**Supplementary Fig. 1**

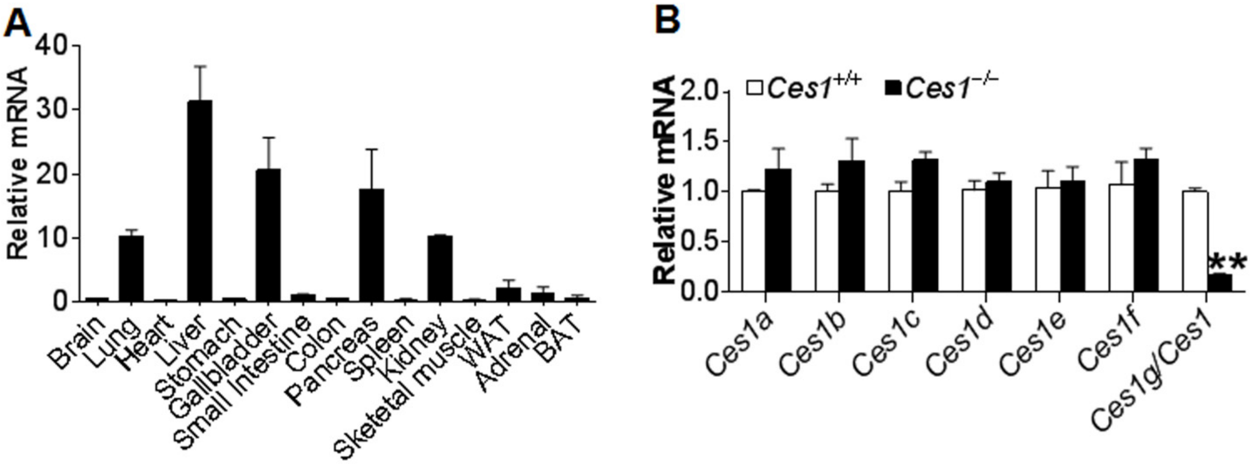

**Supplementary Fig. 2**

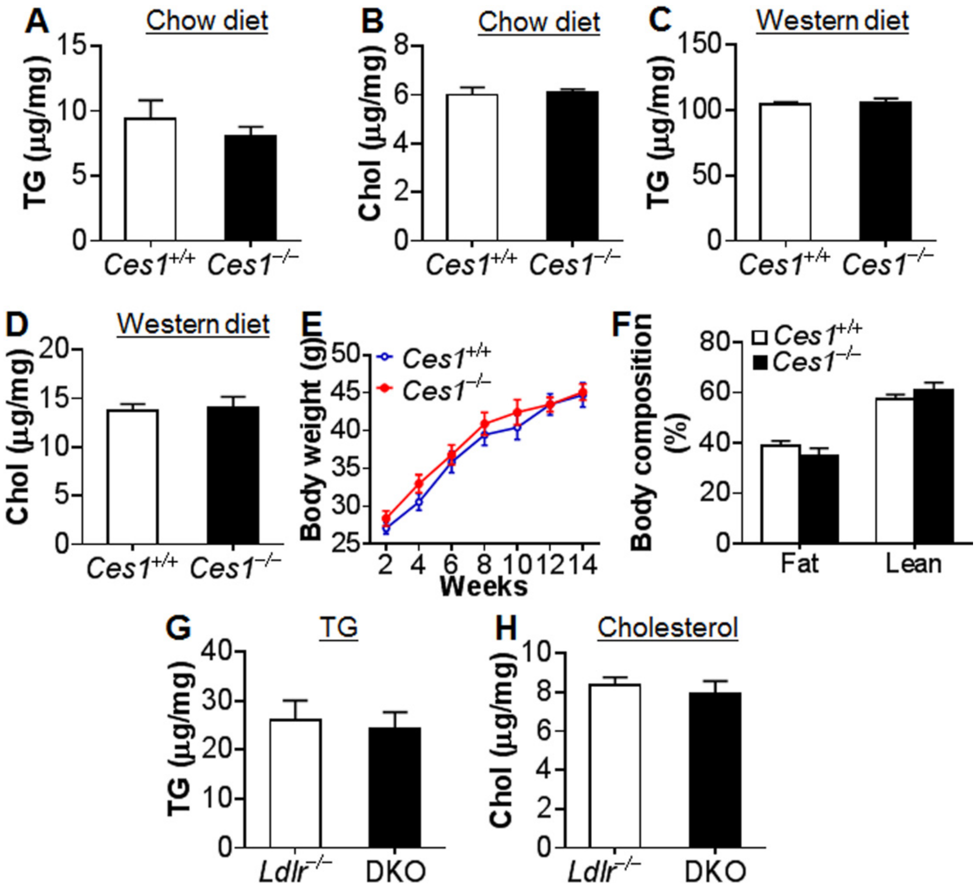

**Supplementary Fig. 3.**

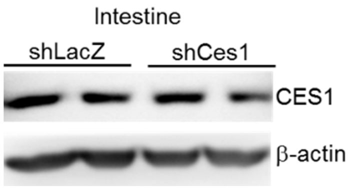

**Supplementary Fig. 4**

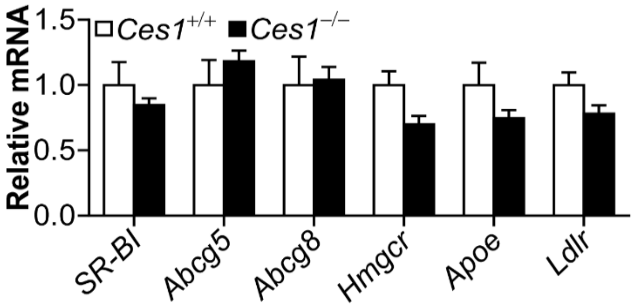

**Supplementary Fig. 5**

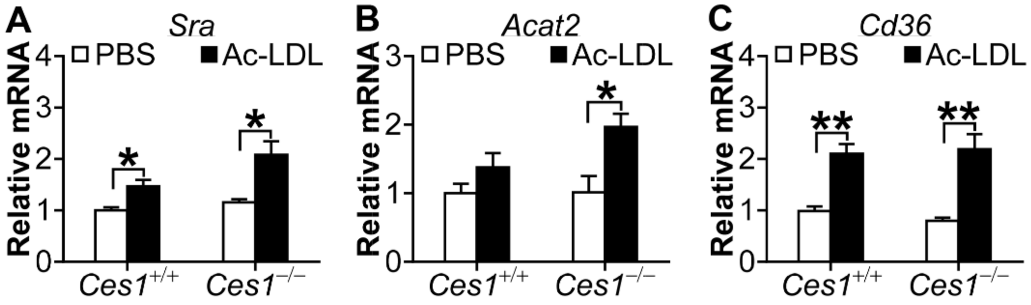

**Supplementary Fig. 6**

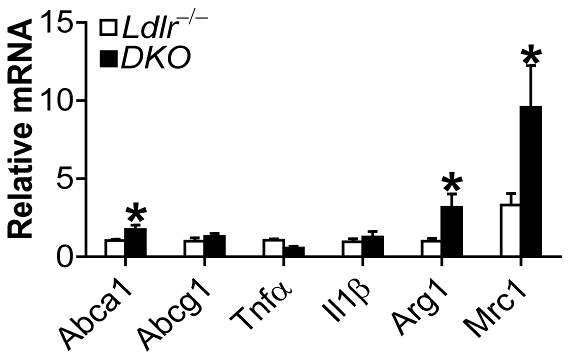

## Original gel/blots

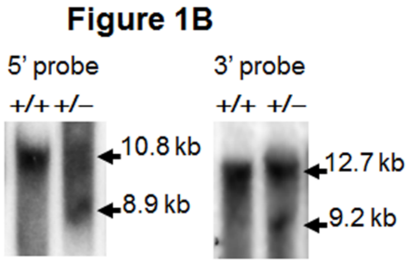


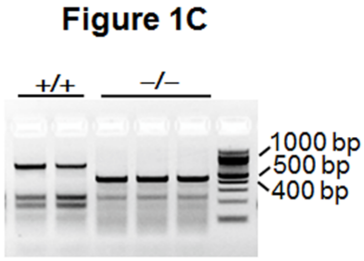


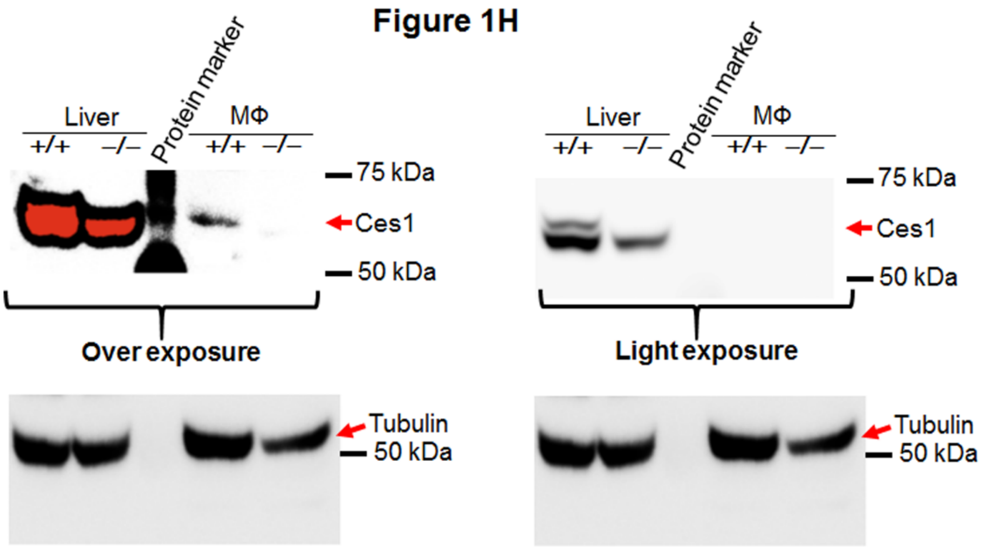


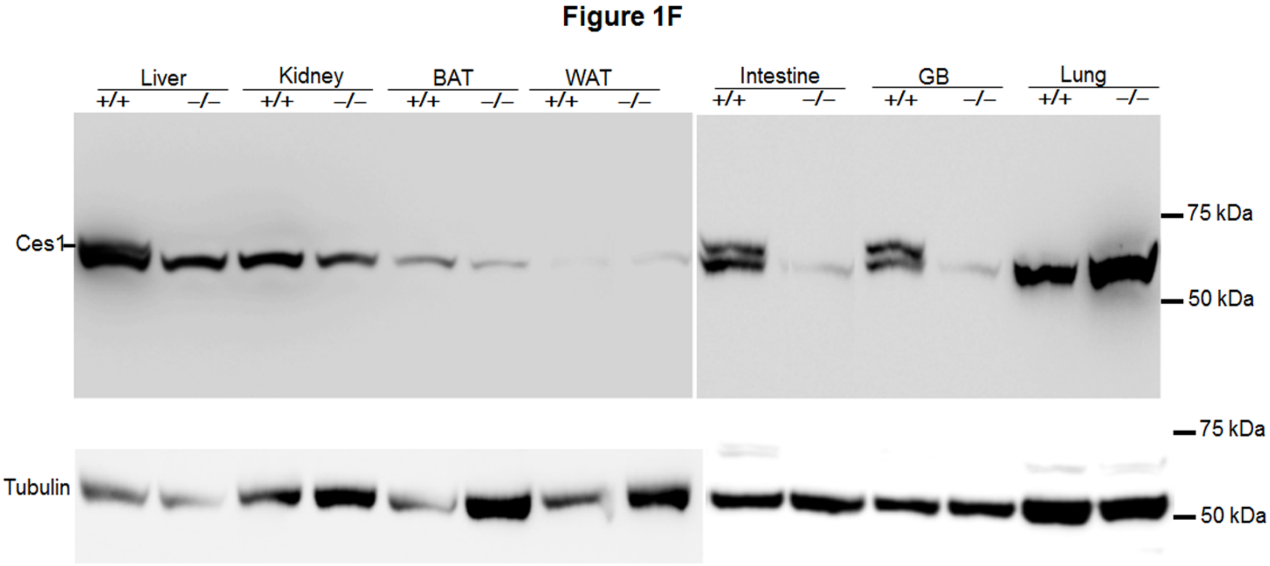

**Figure 3A**

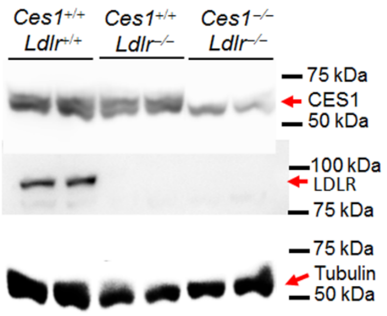


**Figure 4A**

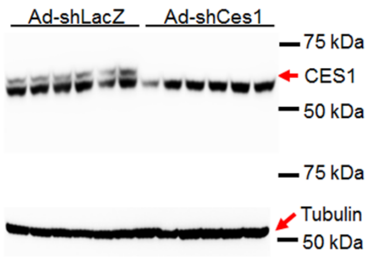


**Suppl Fig. 3**

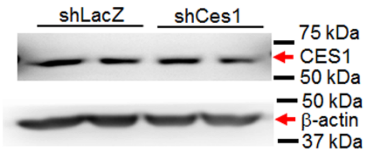

Supplement: Supplementary file 1 — Supplementary Information [file 41598_2017_18232_MOESM1_ESM.pdf]
